# Effects of Virtual Reality Hypnosis on Pain and Anxiety in Oncology Patients During Port-a-Catheter Placement Procedure: A Pilot Study

**DOI:** 10.3390/brainsci16040384

**Published:** 2026-03-31

**Authors:** Yanis Mouheb, Mélanie Louras, Jean-François Maillart, Olivia Gosseries, Claudia Charry, Aminata Bicego, Audrey Vanhaudenhuyse

**Affiliations:** 1 Conscious Care Lab, GIGA Consciousness, GIGA Institute, University of Liège, 4000 Liège, Belgium; yanis.mouheb@doct.uliege.be (Y.M.); ogosseries@uliege.be (O.G.); abicego@uliege.be (A.B.); 2 NeuroRecovery Lab, GIGA Consciousness, GIGA Institute, University of Liège, 4000 Liège, Belgium; melanie.louras@uliege.be; 3 Centre Hospitalier Régional de Huy, 4500 Huy, Belgium; jeanfrancois.maillart@chrh.be (J.-F.M.);; 4 Coma Science Group, GIGA Consciousness, GIGA Institute, University of Liège, 4000 Liège, Belgium; 5 Cognitive Psychology Unit, Social Sciences Faculty, Leiden University, 2333 Leiden, The Netherlands; 6 Interdisciplinary Algology Center, University Hospital of Liège, 4000 Liège, Belgium

**Keywords:** virtual reality hypnosis, PAC placement, anxiety, procedural pain, absorption, dissociation

## Abstract

**Background**: Port-a-catheter (PAC) placement is a common procedure in oncology that, despite local anaesthesia, can induce patient discomfort, procedural pain, and anxiety. Virtual reality hypnosis (VRH), combining immersive virtual reality with clinical hypnosis, has been proposed as a non-pharmacological adjunct to reduce peri-procedural distress. **Objectives**: This pilot study aimed to explore the suitability of VRH during PAC placement and its potential effects on pain, anxiety, and VRH-related experiences, while investigating psychological variables associated with VRH engagement. **Methods**: In this single-arm interventional monocentric prospective pilot study, twenty oncology patients undergoing first-time elective PAC placement received a VRH intervention delivered via a medical-grade head-mounted display throughout the procedure. Pain, anxiety, and VRH-related dimensions—including absorption, dissociation, automaticity, arousal, and sense of presence—were assessed pre- and post-procedure using self-reported numerical rating scales and questionnaires. Non-parametric Wilcoxon tests evaluated pre–post changes, and correlational analyses (Pearson’s and Spearman’s when necessary) explored associations between variables. **Results**: VRH was well tolerated by most participants, although three patients required additional pharmacological support, and four could not complete the session due to intolerance or technical issues. Anxiety scores decreased significantly following VRH, whereas pain showed a non-significant trend toward reduction. Post-procedural absorption and dissociation were positively associated with presence, and higher absorption traits were linked to greater immersive engagement and prior VR/hypnosis experience. Cybersickness was negatively associated with absorption. Older age was correlated with lower post-procedural pain, and females reported higher state anxiety. **Conclusions**: In this pilot, VRH was feasible, well tolerated, and associated with a significant exploratory reduction in procedural state anxiety. Given the single-arm design, these findings constitute directional evidence warranting controlled trial evaluation rather than proof of efficacy. These preliminary results support the rationale for randomised controlled trials to evaluate VRH efficacy, underlying mechanisms, and potential role as a non-pharmacological adjunct in oncology perioperative care.

## 1. Introduction

In oncology care, port-a-catheter (PAC) placement is commonly performed as part of the treatment pathway to facilitate chemotherapy administration [1]. The PAC procedure involves a small incision through which the port device is inserted. In turn, the port is connected to a catheter to allow for direct venous access. The PAC system allows patients to receive the treatment comfortably over extended periods, as it reduces the amount of needle puncture pain while delivering a reliable and long-lasting vascular access for chemotherapy. However, despite its clinical benefits, PAC placement presents several challenges. The procedure is typically performed under local anaesthesia, sometimes with additional conscious sedation. However, it is still associated with patient-reported discomfort, acute pain, and peri-procedural anxiety [2,3,4]. Perioperative anxiety in oncology patients is not inconsequential. Although the most robust evidence linking anxiety to immunological suppression, increased analgesic requirements, and adverse postoperative behavioural outcomes such as kinesiophobia derives primarily from studies of major surgical procedures, the neuroendocrine stress response underlying these associations—including HPA axis activation and sympathoadrenal arousal—can be triggered by anticipatory anxiety independently of procedural invasiveness [5,6,7,8,9,10,11,12,13]. In the oncology context, where anxiety frequently precedes the index procedure, the psychological burden of brief interventions such as PAC placement may be additive rather than isolated, and should not be trivialised on the basis of procedural brevity alone [14,15]. Although the short-term benefits of pharmacological strategies for pain and anxiety management seem quite effective, the adverse effects they may entail, such as nausea, cardiovascular risk, or even increased painkiller consumption [16], indicate the urgent need for safe, cost- and time-effective complementary approaches.

In this framework, virtual reality hypnosis (VRH) appears as a promising adjunct to conventional drug-based therapeutic options. VRH combines the properties of both clinical hypnosis and contemplative virtual reality. Hypnosis is usually defined as “a state of consciousness involving focused attention and reduced peripheral awareness characterised by an enhanced capacity for response to suggestion” [17]. The process allowing one to reach the aforementioned state involves a sequence of procedures that generally starts with an induction aimed at minimising attention to external stimuli, focusing attention on the practitioner’s suggestions, and enhancing responsiveness to suggestions intended to modulate experiences across multiple domains, including emotional, cognitive, and perceptual processes [18]. Evidence from a small but growing number of studies suggests that hypnosis may reduce pain and anxiety during vascular access procedures, including PAC placement [19,20,21]. However, the evidence base specific to this procedure remains limited, and the magnitude of effects varies substantially across individuals as a function of hypnotic suggestibility and therapist expertise. A recent meta-analytic update confirmed a significant analgesic and anxiolytic effect of hypnosis across invasive medical procedures overall, while underscoring high heterogeneity [22]. Standardised delivery via VRH is partly motivated by the aim to reduce reliance on individual suggestibility and to enable more reproducible implementation. However, its efficiency is still highly determined by the individual’s hypnotic suggestibility (responsiveness to hypnotic suggestions) and the hypnotherapist’s skills (e.g., training in clinical hypnosis, relevant knowledge, and experience regarding the patient’s pathology) [23]. From a clinical implementation standpoint, VRH offers the advantage of delivering standardised hypnotic suggestions, which limits the constant need for a trained hypnotherapist. This addresses a major constraint to the widespread adoption of hypnosis in medical settings, namely its reliance on specialised personnel and inter-individual variability in delivery and responsiveness [23,24].

Hypnosis is characterised by several dimensions: absorption, dissociation, automaticity, suggestibility, and reduced arousal [25,26,27,28,29]. The term absorption is used to describe one’s ability to be completely immersed in the experience and is a predictor for hypnotic suggestibility [25,30]. Dissociation is a detachment from bodily or sensory experience, a hallmark of hypnotic analgesia [31,32,33]. Automaticity [29,34,35] is a characteristic of the suggested responses that are believed to be involuntary. Suggestibility is the tendency to conform to instructed suggestions and to suspend one’s critical judgement [36], whereas reduced arousal represents the relaxation and lowering of stress responses that constitute some of the facilitators of the analgesic process [37,38]. Each of these constructs has an independent empirical and theoretical literature; however, their status as fully distinct, separable, and equivalently meaningful clinical mechanisms is not universally agreed upon in the field. In the present study, these dimensions are operationalised as exploratory variables to probe whether VRH engages phenomenological processes theoretically associated with hypnotic responding, rather than as a definitive mechanistic taxonomy.

On the other hand, virtual reality (VR) is “a three-dimensional computer-generated simulated environment, which attempts to replicate real-world or imaginary environments and interactions” [39]. VR has exhibited its efficacy in reducing both procedural pain and anxiety through the creation of a feeling of presence and immersive distraction [40,41,42,43]. Moreover, the concepts of presence [44,45] and immersive propensity [46,47] will be considered, given their known association with VR efficacy, while cybersickness [48,49] will be a subject of assessment as a potential adverse effect. Prior experience with hypnosis or VR will be explored as well.

An important conceptual distinction must be acknowledged between VR-mediated attentional distraction and hypnosis-specific effects. VR operates primarily through attentional capture and sensory redirection [50], redirecting cognitive resources away from nociceptive and anxiogenic stimuli. Clinical hypnosis additionally involves structured suggestion-based modulation of expectations, perceptual appraisal, and voluntary/involuntary distinctions in response generation [32,51]. Whether a standardised VRH protocol can replicate these hypnosis-specific processes in the absence of a live therapist remains an open empirical question, and no claim of functional equivalence is made here. The present study treats VRH as a technology-mediated approach that may engage hypnotic-like processes and examines this possibility through validated hypnosis-related measures, while acknowledging that definitively isolating a hypnotic effect from immersive distraction requires a VR-only comparator arm.

Despite the promise of VRH, the literature remains sparse. Research on adding VRH to treatment for acute pain and anxiety has been conducted only in a few cases, and the findings appear inconsistent [52,53,54,55,56,57,58]. One substantial result was that VRH significantly reduced both pain and anxiety during a pacemaker placement procedure [59] (randomised controlled trial including 61 patients). Also, in a retrospective study, 20 patients undergoing bronchoscopy [60] showed a significant decrease in anxiety. In light of this, the current research aims to evaluate the effect of VRH on both pain and anxiety in oncology patients undergoing PAC placement, while probing into the psychological mechanisms responsible for the potential efficacy of VRH by screening out absorption, dissociation, automaticity, arousal, presence, cybersickness, and prior experience with VR or hypnosis. This pilot study serves as a stepping stone to develop knowledge on the VRH mode of action on patients undergoing a PAC placement, and in the future, to transform such knowledge into monitoring tools that can be used in daily clinical practice. Secondary objectives aim at identifying factors that influence the dissociative effect of VRH, such as absorption, immersive abilities, dissociation traits, and anxiety levels. Further, we hypothesise that VRH induces measurable dissociation; dissociative trait variances should positively impact dissociation scores; and that anxiety traits should impact dissociation scores negatively.

## 2. Materials and Methods

### 2.1. Population

Oncology patients scheduled for elective PAC placement at Huy Regional Hospital were considered for inclusion. Eligibility was determined during the routine preoperative consultation. Inclusion criteria included: age ≥ 18 years, confirmed cancer diagnosis requiring first PAC placement, native French speakers, and provision of written informed consent. Exclusion criteria included: second PAC placement, claustrophobia, general anaesthesia indication, anxiolytics and analgesics use during PAC placement, cognitive impairment, psychiatric disorders, known susceptibility to seizures, or contraindications to VR headset use (e.g., significant visual, auditory and vestibular impairments), chronic pain (visual analogue scale score > 3/10 for more than 6 months) and regular use of painkillers (more than 15 days per month, >3 months). Patients were consecutively screened and enrolled if they fulfilled all inclusion criteria.

This monocentric prospective interventional pilot study was conducted at the Regional Hospital Centre of Huy (Belgium). The first patient was enrolled on 17 February 2023, and the last one on 26 September 2023.

### 2.2. Procedure

At the preoperative consultation, the surgeon (J.-F. M., one of the co-authors) explained both the PAC placement procedure and the associated study. Patients meeting the inclusion criteria were invited to participate, and written informed consent was obtained at that moment. PAC placement was scheduled approximately one week later. On the day of PAC placement, patients arrived at the hospital one hour before the procedure to complete baseline questionnaires (see Data collection tools section). They were then transferred to the operating room, where research staff and the surgical team collaborated to integrate the VRH device into the surgical procedure. Pain and anxiety NRS ratings were collected at two time points: (1) prior to surgical preparation and VRH headset placement (pre-procedural baseline), and (2) in the recovery room following completion of PAC placement and VRH headset removal (post-procedural). It should be noted that the pre-procedural pain NRS reflects the patient’s resting baseline pain level and does not capture procedural pain during the intervention itself. Similarly, the post-procedural NRS is obtained after procedure completion, when procedural nociception would be expected to have been resolved. Accordingly, the pre/post pain comparison reflects a change in pain state between two resting time points rather than intra-procedural analgesic efficacy. Intra-procedural pain assessment was not performed, as interrupting the VRH session to administer ratings would be conceptually counterintuitive and disruptive to the attentional absorption underpinning the intervention’s hypothesised mechanism of action. Following the procedure, patients completed additional post-procedural questionnaires in the recovery room. The study protocol did not incorporate planned co-administration of anxiolytic or analgesic agents alongside VRH. However, rescue pharmacological support (analgesia or sedation) remained available intraoperatively at the discretion of the surgical team if patient safety or comfort required it. Patients who received rescue medication were excluded from the primary per-protocol analysis, consistent with the study’s pre-specified exclusion of planned analgesic or anxiolytic co-use. These cases are reported as protocol deviations.

### 2.3. Intervention

**Figure 1:**
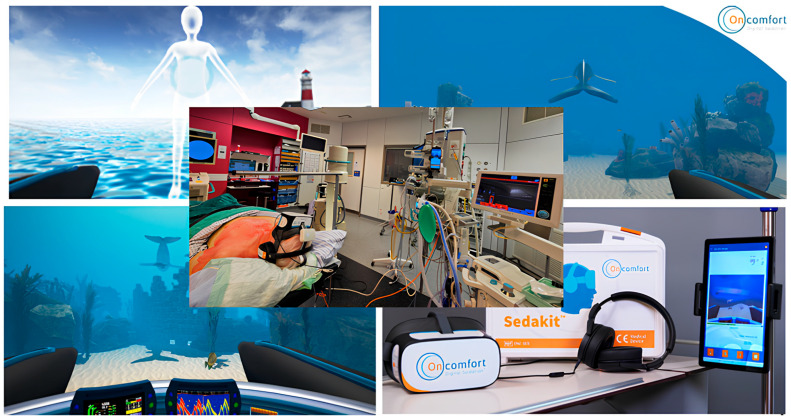
VRH environment (Aqua+© 120 Version 1.1, HypnoVR SAS, Strasbourg, France) representing various phases of the hypnotic session: (**upper left corner**) induction, (**upper right corner**) guidance, and (**lower left corner**) deepening. The user is guided by a whale in a submarine environment and is brought back to the surface at the end of the VRH experience. The experiment took place in a clinical setting, (**centre of the figure**) by means of the X1 Sedakit device, (**lower right corner**).

The intervention consisted of a VRH session delivered via a medical-grade head-mounted display (Sedakit™ X1, HypnoVR SAS, Strasbourg, France). The X1 Sedakit is a non-invasive Class Ia medical device (Directive 93/42/EEC) equipped with a 4K display and a 75 Hz sampling rate. Patients underwent a VRH session using the Aqua+© 120 (Version 1.1) VR software, which provides a standardised clinical hypnosis script designed to reduce anxiety and pain during medical procedures. The VRH session consists of four predefined phases (Figure 1): (1) a hypnosis-based induction phase during which the patient enters the virtual environment, (2) a guidance phase, (3) a deepening phase promoting relaxation, and (4) a re-alerting phase during which the subject is gently brought back to reality. During the session, patients experienced an underwater-themed immersive environment accompanied by synchronised hypnotic narration delivered through high-quality headphones, along with a background light ethereal music, ensuring that patients were both visually and auditorily isolated from the operating room.

A trained member of the research team initiated and monitored each VRH session. A standard VRH duration was selected in advance to correspond to the typical length of a PAC placement (about 20 min). However, the guidance phase could be pragmatically adapted in real time using the device interface. When procedures exceeded the expected duration, additional VRH time was added. Likewise, the session was shortened when the procedure concluded ahead of schedule. Thus, although the induction and resurfacing phases followed the standardised VRH structure, the total duration of VRH exposure varied across patients. VRH was delivered continuously throughout the PAC placement procedure, and the headset was removed after completion. Any adverse event or intolerance (e.g., claustrophobia, cybersickness) was documented and managed intraoperatively and could lead to exclusion from analysis when appropriate.

### 2.4. Data Collection Tools

- Trait measures (pre-procedural questionnaires):

Trait absorption: the Tellegen Absorption Scale (TAS) [25] consists of 34 true/false items, with a dispositional tendency to experience episodes of deep involvement engaging the participant’s cognitive and attentional resources. The total score range is from 0 to 34. Higher scores indicate a greater propensity for absorption.

Trait and state anxiety: the State-Trait Anxiety Inventory (STAI-Y) [61] has two parts: one targeting trait anxiety and the other, state anxiety. Each part consists of 20 items with 4 response options (1 = almost never, 2 = sometimes, 3 = often, 4 = almost always). Total scores can range from 20 to 80, with higher scores indicating a higher level of anxiety.

Trait dissociation: the Dissociative Experience Scale (DES) [62] is a self-report questionnaire assessing the frequency and types of dissociative experiences in everyday life. It consists of 28 items rated on a numerical rating scale (from 0% = 0 percent of the time to 100% = 100% of the time). The total score ranges from 0 to 100. Although a cutoff score of 45 has been proposed to suggest a dissociative disorder, the DES is not a diagnostic instrument. Rather, it serves as a screening tool indicating when further clinical assessment may be needed.

Immersive propensity: the Immersive Tendencies Questionnaire [46,47] assesses several factors associated with immersive tendencies, including the tendency to become involved in activities, the tendency to maintain focus on current activities, and the tendency to engage in video game play. The higher the score, the more one is immersed in the virtual environment.

- Pre-procedural questions: prior experience with hypnosis or VR was assessed with yes/no questions, and demographic characteristics (age, biological sex) were gathered from patients’ files.
- VRH State Measures

Pain intensity was measured with a 0–10 Numerical Rating Scale (NRS) before and after PAC placement. Patients were asked, “On a 0–10 scale, could you assess your perception of pain? 10 meaning the worst imaginable pain and 0 meaning no pain.”

Anxiety was measured with a 0–10 NRS pre- and post-procedure. Patients were asked, “On a 0–10 scale, could you assess how anxious you feel? 10 meaning the most imaginable anxiety and 0 meaning no anxiety.”

Absorption was assessed with a 0–10 NRS post-procedure. Patients were asked, “On a 0–10 scale, could you assess to what extent did you feel absorbed and experience your attention as focused on the experience you were undergoing? 10 meaning completely absorbed and 0 meaning not at all absorbed in the VRH experience.” [30]

Dissociation was assessed using a post-procedural 0–10 NRS. Patients were asked: “On a 0–10 scale, did you experience a dissociation between your bodily sensations and the real environment, with 0 meaning that you felt fully grounded in the room and 10 meaning that you were completely immersed in a subjective experience, totally disconnected from the here and now?” [30]

Automaticity was assessed using a 0–10 NRS post-procedure. Patients were asked: “On a 0–10 scale, to what extent did you feel in control of your actions and your thoughts, with 0 meaning a perfect sense of control, where you felt like the agent and driving force behind your actions and thoughts and 10 meaning that you felt like a passive witness to your actions and thoughts, which arose and unfolded naturally without a sense of being their originator?” [29]

Wakefulness/arousal was assessed using a 0–10 numerical rating scale (NRS). Patients were asked: “On a 0–10 scale, to what extent did you feel aroused, with 0 meaning not aroused at all and 10 meaning extremely aroused?”

Presence was assessed with the Presence Questionnaire [44,45], via a lab-made self-reported questionnaire used to evaluate four sub-categories: spatial presence, ecological validity, social presence and copresence the patient could experience during the VRH experience. It consists of 12 items, each of them with a 7-point response option (ranging from 0 = I do not agree at all to 7 = I fully agree).

Cybersickness was assessed with the Cybersickness Questionnaire (CSQ) [49], a 16-item self-reported questionnaire used to evaluate participants’ sickness levels after immersions in VRH. Participants evaluated the severity of each of their symptoms (e.g., dizziness, nausea, vertigo) on a 4-point Likert scale (ranging from 0 = none to 3 = severe). All raw items were summed in order to obtain a total score; a lower total score refers to less severe sickness.

### 2.5. Statistical Analyses

Descriptive statistics summarise demographic and clinical characteristics. Normality was assessed visually and using the Shapiro–Wilk test. Given the small sample size and the non-normal distribution of most ratings, non-parametric Wilcoxon signed-rank tests for paired samples were used for all comparisons between pre- and post-procedural measures. Secondary analyses assessed the relationships between psychological variables (absorption, dissociation, automaticity, arousal, presence), subjective pre- and post-procedural ratings of pain and anxiety, and pre- and post-procedural questionnaires (psychological and VRH-related). The Pearson correlation coefficient (r) was employed as an indicator to evaluate how some variables could have influenced the outcomes of the pre- and post-procedural measures and how they could impact on one another. When distributions deviated from normality, we used a Spearman rank-order correlation coefficient (ρ), the non-parametric equivalent of Pearson’s r. All correlation tests were two-tailed, testing for the presence of both positive and negative associations. Prior experience with VR or hypnosis and biological sex were treated as binary variables for statistical analyses. Given the exploratory nature of this pilot study, no correction for multiple comparisons was applied, consistent with established guidance for hypothesis-generating research [63,64]. All correlation findings should be interpreted as directional signals for future confirmatory research. Effect size magnitudes are reported and interpreted according to the following thresholds for individual-differences research and pain contexts: |r| < 0.10 = negligible; 0.10–0.29 = small; 0.30–0.39 = moderate; ≥0.40 = large [65,66]. Narrative discussion focuses on findings with at least moderate effect sizes (|r| ≥ 0.30) in conjunction with *p* < 0.05. All Pearson’s r and Spearman’s rho values are reported with 95% confidence intervals. A significance threshold of *p* < 0.05 was applied. Statistical analyses were performed using JASP statistical software (version 0.95.3) [67]. The primary analysis population was defined a priori as a per-protocol completer set comprising patients who completed the full VRH session without requiring intraoperative rescue pharmacological support (*n* = 20). Patients who did not complete the VRH session (*n* = 4) or who required intraoperative rescue medication (*n* = 3) were excluded from the primary analysis. No intention-to-treat analysis was conducted, given the single-arm exploratory design and non-inferential objectives of this pilot study.

## 3. Results

### 3.1. Participant Flow

**Figure 2:**
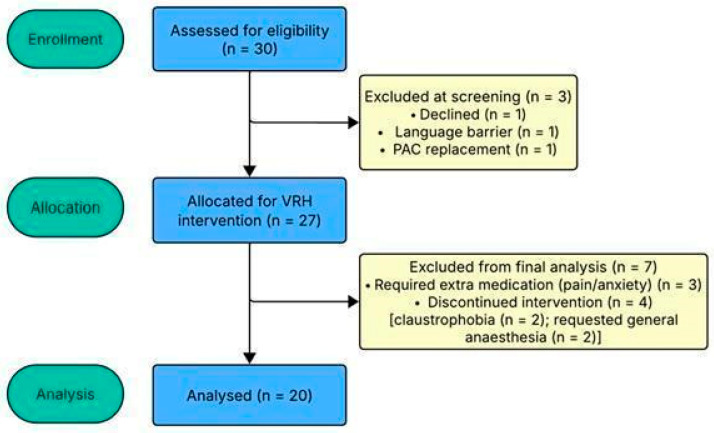
CONSORT flow diagram of participant progression through the study.

A total of 30 oncology patients scheduled for PAC placement were assessed for eligibility. Three were excluded during screening: one declined participation for religious reasons, one was unable to participate due to insufficient language proficiency, and one was scheduled for a PAC replacement rather than a primary placement. Among the remaining 27 patients, three required additional pharmacological support during the procedure (morphine for pain or benzodiazepines for anxiety), and four were unable to complete the VRH exposure as planned due to technical difficulties or intolerance. Specifically, two patients reported severe claustrophobia preventing headset use (a response contingent on physical device contact and not reliably predictable at preoperative consultation), and two requested general anaesthesia prior to or during the procedure. Consequently, 20 patients completed the VRH intervention (completion rate: 74%) and were included in the final analyses (Figure 2, CONSORT diagram). No serious adverse events were recorded. The completion rate of 74% is consistent with comparable VR clinical studies in procedural oncology settings [40,43]. Correlations between pre-procedural questionnaires (absorption trait, anxiety trait and state, dissociation trait and immersive propensity) and the other variables were conducted on 19 patients, as data were missing for one patient.

Demographic characteristics are described in Table 1.

Pre- and post-procedural measures comparisons.

**Figure 3:**
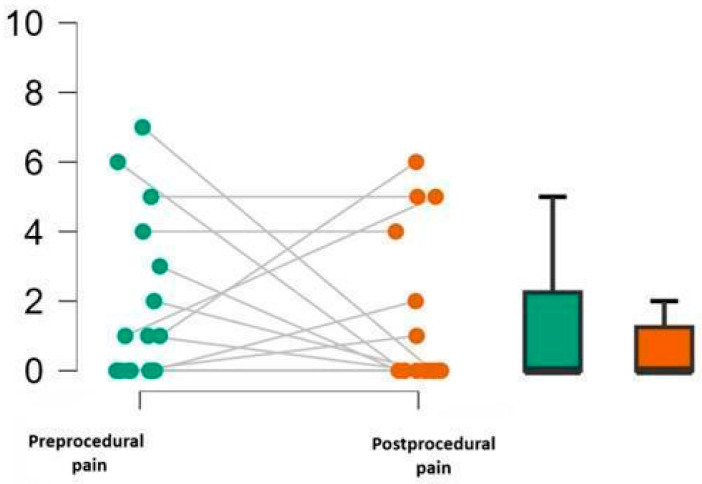
Pre- and postprocedural pain scores. Individual pain ratings before and after PAC placement with VRH are displayed (**left panel**), with paired observations connected by grey lines. Boxplots (**right panel**) illustrate median and interquartile range; whiskers represent minimum and maximum values.

Pain intensity (Figure 3) showed a non-significant trend towards reduction across the sample following the VRH intervention (W = 27.000, z = 0.533, *p* = 0.317), with a median reduction of 1.0 point (Hodges–Lehmann estimate), 95% CI [−2.0, +∞]. Although interindividual variability was present, most patients reported low post-procedural pain compared to rather similar baseline pain levels (cf. Supplementary Table S1).

**Figure 4:**
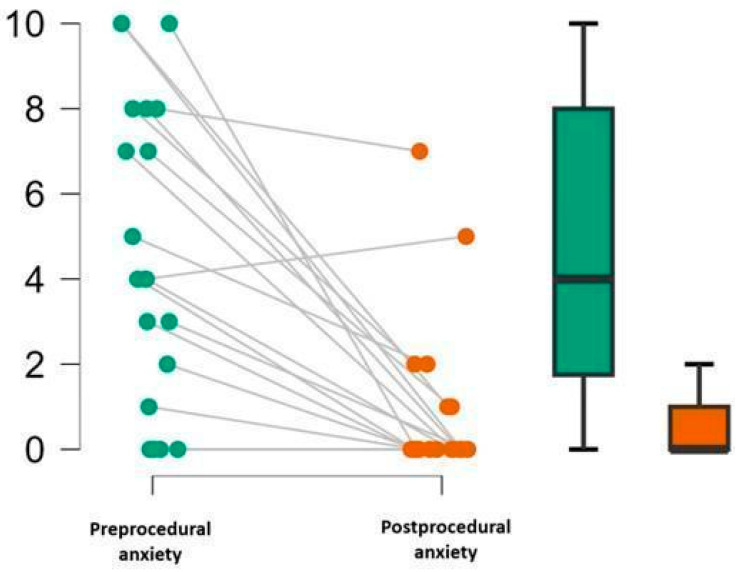
Pre- and postprocedural anxiety scores. Individual anxiety ratings before and after PAC placement with VRH are displayed (**left panel**), with paired observations connected by grey lines. Boxplots (**right panel**) illustrate median and interquartile range; whiskers represent minimum and maximum values.

Anxiety levels (Figure 4) significantly decreased after the VRH session (W = 134.000, z = 3.413, *p* < 0.001), with a median reduction of 4.5 points (Hodges–Lehmann estimate), 95% CI [3.0, +∞].

### 3.2. Correlation Testing

All correlation analyses are exploratory and should be treated as directional hypotheses for future confirmatory investigation. No correction for multiple comparisons was applied, consistent with the hypothesis-generating objectives of this pilot study [63]. Effect sizes and 95% confidence intervals are reported for all Pearson’s r values. Narrative interpretation is restricted to associations with *p* < 0.05 and |r| or |ρ| ≥ 0.30 (moderate effect size minimum threshold) [65].

Results indicate that state absorption significantly positively correlated with presence (r = 0.573, z = 0.653, SE = 0.243, 95% CI [0.248, 1.000], *p* = 0.004) and state dissociation (ρ = 0.564, z = 0.639, SE = 0.246, 95% CI [0.235, 1.000], *p* = 0.005) on the one hand, and negatively correlated with cybersickness (ρ = −0.535, z = −0.597, SE = 0.245, 95% CI [−1.000, −0.195], *p* = 0.008) and post-procedural anxiety (ρ = −0.592, z = −0.680, SE = 0.247, 95% CI [−1.000, −0.274], *p* = 0.003).

Trait absorption was significantly positively correlated with presence (r = 0.643, z = 0.764, SE = 0.250, 95% CI [0.339, 1.000], *p* = 0.001), immersive propensity (r = 0.579, z = 0.661, SE = 0.250, 95% CI [0.245, 1.000], *p* = 0.005), prior experience with VR (ρ = 0.408, z = 0.433, SE = 0.250, 95% CI [0.022, 1.000], *p* = 0.041), and with prior experience with hypnosis (ρ = 0.408, z = 0.433, SE = 0.250, 95% CI [0.022, 1.000], *p* = 0.041).

State anxiety (STAI) differed significantly by sex (r = 0.526, z = 0.584, SE = 0.250, 95% CI [0.172, 1.000], *p* = 0.010), with higher scores observed in females.

Moreover, cybersickness was significantly positively correlated with prior experience with hypnosis (ρ = 0.508, z = 0.560, SE = 0.252, 95% CI [0.148, 1.000], *p* = 0.013), trait dissociation (ρ = 0.397, z = 0.420, SE = 0.249, 95% CI [0.009, 1.000], *p* = 0.046) and post-procedural anxiety (ρ = 0.448, z = 0.482, SE = 0.244, 95% CI [0.083, 1.000], *p* = 0.024).

State dissociation was significantly positively correlated with presence (r = 0.632, z = 0.745, SE = 0.243, 95% CI [0.333, 1.000], *p* = 0.001).

Presence was significantly positively correlated with immersive propensity (ρ = 0.396, z = 0.419, SE = 0.249, 95% CI [0.008, 1.000], *p* = 0.047).

Wakefulness/arousal was significantly positively correlated with state anxiety (r = 0.430, z = 0.460, SE = 0.250, 95% CI [0.049, 1.000], *p* = 0.033).

It should also be noted that post-procedural pain was significantly negatively correlated with age (ρ = −0.387, z = −0.408, SE = 0.242, 95% CI [−1.000, −0.009], *p* = 0.046).

Only associations with *p* < 0.05 and |r| or |ρ| ≥ 0.30 are interpreted in the narrative. All remaining associations are reported in Supplementary Table S2 for completeness. Effect sizes and 95% confidence intervals are provided for all Pearson’s r and Spearman’s rho values. No other correlation was deemed noteworthy; a full account of Shapiro–Wilk test results is provided in Supplementary Table S3.

## 4. Discussion

This pilot study is the first of its kind to explore VRH during PAC placement in oncology while also evaluating psychological measures related to hypnosis and VR, yielding a significant anxiety reduction signal alongside theoretically coherent associations among hypnotic dimensions. These findings are appropriately interpreted as exploratory: without a control condition, the contributions of expectancy, staff attention, and natural post-procedural relief cannot be quantified. We do not claim efficacy; we report a signal of sufficient magnitude and internal coherence to motivate a fully powered controlled trial. The correlational findings reported here are exploratory and must not be interpreted as mechanistic evidence. They represent theoretically coherent patterns of moderate-to-large effect size that constitute the primary hypothesis-generating output of this pilot study. Their replication and extension in a larger controlled study are required before any mechanistic conclusions can be drawn.

The statistically significant reduction in state anxiety (median 4.5 points) is consistent with a beneficial effect of VRH on peri-procedural anxiety. However, in the absence of a control condition, this reduction cannot be causally attributed to the VRH intervention. The natural resolution of anticipatory anxiety following procedure completion is a plausible alternative explanation, and the relative contributions of VRH-specific mechanisms, expectancy, and post-procedural relief cannot be disentangled in the framework of this study. Nonetheless, a reduction of this magnitude—nearly half the available scale range in a population with moderate pre-procedural anxiety—is difficult to dismiss as clinically negligible, and its convergence with theoretically coherent associations among hypnotic dimensions lends it additional interpretative weight. These findings constitute a meaningful exploratory signal warranting investigation in a controlled design.

The non-significant pain trend (W = 27.000, *p* = 0.317, median reduction = 1.0 NRS point) must be interpreted in the context of the measurement design: both time points captured resting pain levels rather than peak procedural nociception. Pre-procedural pain was already low, limiting the range of possible reduction. Furthermore, intra-procedural pain assessment was not feasible in this context: interrupting an immersive VRH session to administer pain ratings would probably break the attentional absorption and modified awareness that constitute the intervention’s core mechanism, rendering such measurement both disruptive and conceptually contradictory [54,68]. These factors preclude interpretation of the pre/post pain comparison as a measure of intra-procedural analgesic efficacy. However, this neither deflates the central role of pain and anxiety assessment in such a clinical context, nor the potential adjunctive value of VRH as a complementary, non-pharmacological approach to standard care, but rather highlights the need for controlled studies. Future studies should incorporate a randomised controlled design with at minimum a standard-care comparator arm, to isolate the specific contribution of the VRH component and enable meaningful between-group inference on both pain and anxiety outcomes.

It should further be noted that several of the reported confidence intervals for correlation coefficients reach the mathematical boundary of ±1.000, a consequence of the small sample size (*n* = 20) in combination with large observed effect sizes. When back-transformed from Fisher’s z-space to r-space, CIs for large correlations in small samples routinely truncate at ±1, rendering the upper (or lower) bound of these intervals statistically uninformative. This means that while the observed point estimates are directionally meaningful, the true population correlations are estimated with very low precision and could be substantially larger or smaller than the observed r values suggest. This is an additional reason to treat all correlation findings as preliminary and hypothesis-generating rather than as stable effect size estimates [69].

Conversely, the observed significant post-procedural decrease in anxiety offers clinical relevance and seems to be consistent with literature involving hypnosis and VR, whether used independently or combined, as anxiolytic agents in medical procedures [22,41,60]. As stated previously, although pain only shows a non-significant downward trend, anxiety plays a central role in perioperative care [58], as increased anxiety levels can lead to higher requirements in analgesics, transform postoperative recovery into an uphill battle, and negatively affect immune function [5,6]. This therefore supports the relevance of VRH in this context as a valuable non-pharmacological adjunctive strategy to promote peri-procedural anxiolysis. In addition, trait anxiety did not correlate with dissociation, while arousal and state anxiety showed a strong correlation. Anxiety could thus be interpreted as a potential barrier to reaching a deeper hypnotic state, rather than a facilitator [70,71]. This pattern aligns with prior evidence showing that anxiety consumes attentional resources and reduces absorption, which are critical for achieving dissociation in hypnosis [26,72].

Beyond clinical outcomes, analyses of hypnotic dimensions showed that VRH elicited experiential outcomes consistent with clinical hypnotic-like experiences. State absorption was positively correlated with state dissociation and the sense of presence, while negatively associated with post-procedural anxiety and cybersickness. The positive associations observed between state absorption, state dissociation, and sense of presence are consistent with—though not confirmatory of—theoretical models in which these dimensions co-occur during hypnotic-like experiential states [25,73,74]. These correlational patterns do not constitute evidence of a qualitatively distinct hypnotic induction and may partly reflect shared variance attributable to general immersive engagement or expectancy. Nevertheless, the specific pattern of the absorption–dissociation coupling and its relationship to lower post-procedural anxiety is theoretically coherent with the neodissociation model of hypnotic analgesia, and is not a standard prediction of VR distraction models alone. These findings are best treated as theoretically interesting exploratory hypotheses. Moreover, the strong presence–dissociation coupling observed in this study suggests that VR environments may facilitate absorption (attentional narrowing) alongside a stronger detachment from one’s physical reality (dissociation), two core components of hypnosis.

Cybersickness remains one of the main limitations of VR interventions, and its negative association with state absorption is therefore particularly noteworthy. The prevailing theoretical account, sensory mismatch, articulates the idea that cybersickness will arise when sensory inputs convey incongruent information [75]. The negative association between cybersickness and absorption is theoretically interpretable within the sensory mismatch framework, wherein higher attentional engagement may reduce conscious processing of vestibular–visual incongruence. However, the present study did not control for technical determinants of cybersickness, including display latency, frame rate, or individual vestibular sensitivity, which are established determinants of symptom severity [75]. The proposed mechanism is therefore speculative and constitutes a theoretically derived hypothesis rather than an empirical conclusion. Future studies should measure these technical parameters alongside individual difference variables. These findings appear consistent with VR literature linking higher attentional engagement and sense of presence to decreased cybersickness [76,77]. For instance, in a recent qualitative study, authors tested the user experience of VRH in myeloma patients who reported low scores of cybersickness-related nausea and oculomotor symptoms [78]. These findings highlight the need to conceptualise the relationship between cybersickness and the sense of presence as non-uniform and more complex than commonly assumed. Although we acknowledge that correlations between the sense of presence and cybersickness are inconsistent in VR literature [77], variability may rely on content, individual responsiveness, test demands and measurement, with the possibility of a two-way relationship (cybersickness reducing the sense of presence rather than vice versa could fuel this construct of bidirectionality). Thus, we would like to stress that current findings regarding higher absorption (both state and trait) and the sense of presence co-occurring with reduced cybersickness are framed as correlational and exploratory, and in no way imply causality or a protective effect.

Two associations warrant particular caution: cybersickness–trait dissociation (ρ = 0.397, 95% CI [0.009, 1.000]) and presence–immersive propensity (ρ = 0.396, 95% CI [0.008, 1.000]). In both cases, the lower confidence bound approaches zero, indicating that the data are statistically compatible with a negligible true association. These findings should be regarded as highly tentative and are not discussed as meaningful signals.

Trait measures also seem to have an impact on VRH outcomes. More particularly, we observed a positive association between trait absorption and the sense of presence, the propensity for immersion, and prior VR/hypnosis experience. Consistent with previous findings, these results suggest a supporting role of dispositional openness to immersive and altered states in facilitating engagement in a hypnotic experience [36,79,80].

The observed negative association between age and post-procedural pain (ρ = −0.387, 95% CI [−1.000, −0.009], *p* = 0.046) is consistent with existing literature on age-related differences in pain appraisal and reporting [81,82]; however, with a confidence interval lower bound approaching zero this association is among the most statistically fragile in the present dataset and should be treated as a hypothesis for future investigation rather than a replicable finding. Moreover, female patients displayed higher state anxiety ratings, which also aligns with well-documented sex differences in anxiety prevalence and perioperative stress responses [83,84], although our small sample size precludes inferential strength.

However, in the absence of a control condition, alternative explanations for the observed anxiolytic effects, including expectancy, novelty, or relief following procedure completion, cannot be ruled out.

The attrition pattern observed provides important implementation guidance. Intraoperative VRH intolerance was primarily attributable to claustrophobic reactions to the head-mounted display, which differ phenomenologically from generalised spatial claustrophobia and may not be captured by standard pre-operative screening questionnaires. One could argue that brief preoperative device familiarisation could theoretically reduce intraoperative intolerance; however, this approach entails a meaningful methodological trade-off. Pre-exposure would alter the very first-session phenomenology that this study sought to characterise, introducing a habituation confound that is difficult to disentangle from the intervention effect itself. Future protocols will need to weigh tolerability optimisation against the integrity of the naive-exposure condition. Patients with strong preferences for general anaesthesia or very high trait anxiety may also benefit from additional pre-procedural preparation or may be suboptimal candidates for VRH alone. The 74% completion rate in this pilot is encouraging; future protocols should define prospective criteria for rescue medication administration to enable transparent completer vs. intent-to-treat reporting.

## 5. Limitations

Given its pilot design, this study is subject to several methodological limitations. First, the single-arm design is the principal methodological limitation of this study and precludes causal attribution of observed changes in anxiety to the sole VRH intervention. In the context of this pilot study, whose declared primary objectives were feasibility and tolerability assessment rather than efficacy evaluation, this design is appropriate and standard [85,86]. Nevertheless, it must be acknowledged that any observed reduction in procedural anxiety is consistent with, but cannot be attributed to, the sole VRH intervention, and alternative explanations, including natural post-procedural anxiety resolution, expectancy, and staff attention, remain plausible. An ongoing multicentre RCT (NCT06883786) is specifically designed to address these questions with appropriate control conditions. This prevents isolation of hypnosis-specific effects from those attributable to immersive distraction per se. Immersive VR environments have independently demonstrated clinically significant reductions in procedural pain and anxiety across a range of settings, including port-a-catheter placement [40,43], and the multicomponent nature of the VRH intervention—combining visual immersion, auditory hypnotic narrative, music, and sensory isolation—means that multiple active ingredients may have contributed to the observed effects. The discussion of hypnotic mechanisms is therefore appropriately framed as exploratory. Nevertheless, we note that the use of dispositional trait absorption (Tellegen Absorption Scale) as a predictor of VRH engagement provides one form of convergent support that is not reducible to VR engagement alone: trait absorption predicts hypnotic suggestibility independently of VR exposure [25,87], and its significant positive association with state presence and immersive propensity in the current data is consistent with, though not confirmatory of, hypnotic engagement mechanisms. Future controlled trials incorporating VR-only, hypnosis-only, and standard-care arms—alongside formal hypnotisability assessment—will be required to disentangle these contributions.

Second, the monocentric nature of the study and the small sample size limit generalisability and statistical power. Further, a common limitation of some interventional single-arm studies is that participants tend to be more motivated, engaged, or have stronger bonds with study staff than patients in usual care, which may influence outcomes and reflect aspects of social desirability [88]. In the present study, we did not assess these variables, and their potential impact on the results cannot be excluded. Last, one could argue that our choice not to correct for multiple comparisons could lead to false positives; however, in line with Bender and Lange’s seminal paper [63], such corrections in exploratory studies may be overly conservative and reduce the ability to detect potentially meaningful effects, so results should instead be interpreted cautiously. The post-procedural assessment of hypnotic dimensions relied on single-item NRS ratings (0–10) rather than validated multi-item psychometric instruments. This represents a deliberate methodological trade-off between measurement fidelity and clinical feasibility: administering multi-item scales to oncology patients in the immediate post-operative period would impose unacceptable respondent burden. Single-item ratings of hypnotic state constructs have precedent in the hypnosis and VRH literature [30,54] and the items employed were grounded in established conceptual definitions; however, their construct validity and reliability relative to multi-item standards cannot be assumed. Future studies should seek to incorporate brief validated instruments adapted for clinical settings.

A further limitation concerns the absence of systematic intervention dose recording. Due to technical constraints in the data logging procedure, VRH session durations were not prospectively captured and cannot be reported retrospectively. While the adaptive nature of the VRH duration was a deliberate and clinically motivated design choice—ensuring continuous coverage throughout a procedure whose length varies across patients—the inability to report actual exposure time represents an incontrovertible limitation of this study. Systematic and prospective recording of intervention dose, including session duration, phase-specific timing, and any device-related interruptions, should be considered a methodological priority in future VRH clinical studies.

Despite these constraints, the consistency of the anxiety reduction and the convergence between clinical outcomes and psychological markers strengthen confidence that VRH seems to engage meaningful psychological processes rather than producing purely epiphenomenal effects. This preliminary study allowed us to pinpoint the aforementioned limitations, which are currently being addressed in an ongoing multicentre randomised controlled trial (ClinicalTrials.gov identifier: NCT06883786). The results derived from this subsequent trial will be essential to confirm efficacy, disentangle underlying mechanisms, and determine whether VRH provides additive benefits beyond standard care or VR-only interventions in surgical oncological settings.

## 6. Conclusions

This pilot study demonstrates that VRH can be feasibly and tolerably integrated into PAC placement workflows in an oncology setting and that it is associated with a significant exploratory anxiety reduction signal and theoretically coherent engagement of hypnotic-like processes. This finding should be interpreted as an exploratory signal rather than evidence of efficacy. The pain reduction trend was non-significant. Associations between absorption, dissociation, and presence provide theoretically coherent but non-confirmatory evidence of hypnotic-like engagement. These findings provide the empirical basis for a randomised controlled trial and suggest that VRH warrants serious evaluation as a non-pharmacological adjunct in oncology perioperative care.

## Figures and Tables

**Table 1:** Demographic characteristics of the effective sample completing the VRH intervention.

Characteristic	Effective Sample Completing VRH (N = 20)
Age, Years	
Mean ± SD	68.5 ± 8.5
Range	56–82
Sex, n (%)	
Female	15 (75.0%)
Male	5 (25.0%)

## Data Availability

The raw data supporting the conclusions of this article will be made available by the authors on request.

## Supplementary Materials

The following supporting information can be downloaded at: https://www.mdpi.com/article/10.3390/brainsci16040384/s1. Table S1: Descriptive statistics for clinical, phenomenological, and demographic variables; Table S2: Full correlation matrix of study variables; Table S3: Shapiro–Wilk test for bivariate normality of all study variables.
