# Supplementary material for: Effects of Virtual Reality Hypnosis on Pain and Anxiety in Oncology Patients During Port-a-Catheter Placement Procedure: A Pilot Study
Source: Brain Sci. 2026 Mar 31;16(4):384. doi: 10.3390/brainsci16040384 (PMC13115483; doi:10.3390/brainsci16040384)
Supplement: Supplementary file 1 [file brainsci-16-00384-s001.zip › brainsci-4194879-supplementary.pdf]

Descriptive Statistics

	Anxiety_pre	Anxiety_post	Pain_pre	Pain_post	Absorption	Dissociation	Wakefulness/arousal	Automaticity	Cybersickness
Valid	20	20	20	20	20	20	20	20	20
Missing	0	0	0	0	0	0	0	0	0
Mean	4.700	0.900	1.500	1.150	6.900	4.600	7.700	3.800	1.700
Std. Deviation	3.585	1.889	2.259	2.059	2.150	3.315	3.729	3.694	3.011
Minimum	0.000	0.000	0.000	0.000	1.000	0.000	0.000	0.000	0.000
Maximum	10.000	7.000	7.000	6.000	10.000	10.000	10.000	10.000	11.000

	Presence	Immersive propensity	STAI_State score	STAI_Trait score	Absorption (trait)	Dissociation (trait)	Age	Sex (F=1, M=0)	Prior_VR use	Prior_hypnosis practice
Valid	20	19	19	19	19	19	20	20	19	19
Missing	0	1	1	1	1	1	0	0	1	1
Mean	41.200	49.158	47.368	39.053	14.947	1.217	68.500	0.750	0.105	0.105
Std. Deviation	18.718	16.621	13.039	6.544	7.692	0.953	8.513	0.444	0.315	0.315
Minimum	17.000	21.000	20.000	26.000	0.000	0.036	56.000	0.000	0.000	0.000
Maximum	84.000	87.000	68.000	52.000	27.000	4.007	82.000	1.000	1.000	1.000

Table S1. Descriptive statistics for clinical, phenomenological, and demographic variables.

This table presents descriptive statistics (valid cases, missing values, mean, standard deviation, minimum, and maximum) for pre- and post-procedural anxiety and pain ratings, post-procedural phenomenological state measures (absorption, dissociation, wakefulness/arousal, automaticity), VR-related variables (cybersickness, presence, immersive propensity), trait-level psychological measures (state and trait anxiety, absorption trait, dissociation trait), and demographic and experiential variables (age, sex, prior VR use, prior hypnosis practice). Anxiety, pain, and phenomenological state variables were assessed using 0–10 numerical rating scales unless otherwise specified. Presence and immersive propensity were assessed using multi-item questionnaires. Sex was coded as a binary variable (female = 1, male = 0), and so were prior VR use and prior hypnosis practice (yes = 1, no = 0).

Variable	Anxiety_pre	Anxiety_post	Pain_pre	Pain_post	Absorption	Dissociation	Wake/arousal	Automaticity	Cybersickness	Presence	Immersive_propensity	STAI_State	STAI_Trait	Absorption_trait	Dissociation_trait	Age	Sex	Prior_VR	Prior_hypnosis
Anxiety_pre	1.00	0.421	0.231	0.181	-0.102	-0.104	0.235	-0.217	0.159	0.054	-0.221	0.653**	0.531*	-0.120	0.205	0.128	0.273	0.032	-0.126
Anxiety_post	0.267	1.00	0.456*	0.642**	-0.592**	-0.314	0.140	0.146	0.448*	-0.348	0.073	0.269	0.266	-0.014	-0.323	-0.016	0.087	0.101	0.222
Pain_pre	0.201	0.284	1.00	0.266	-0.182	0.129	0.338	0.109	-0.127	-0.181	-0.117	0.284	0.377	-0.280	0.071	-0.026	0.187	-0.279	-0.052
Pain_post	0.220	0.708***	0.198	1.00	-0.316	-0.244	-0.035	-0.149	0.389	-0.058	0.230	0.032	0.012	0.237	-0.244	-0.387	0.062	0.141	0.566*
Absorption	-0.134	-0.715***	-0.087	-0.436	1.00	0.564**	0.112	0.101	-0.535*	0.502*	0.215	-0.085	-0.202	0.025	0.375	0.151	0.020	-0.016	-0.240
Dissociation	-0.126	-0.292	0.211	-0.245	0.563**	1.00	0.449*	0.308	-0.316	0.559*	0.308	0.036	-0.323	0.375	1.00	-0.177	0.081	0.048	-0.222
Wake/arousal	0.127	0.108	0.275	0.068	-0.043	0.326	1.00	0.414	-0.169	-0.130	0.047	0.539*	-0.216	-0.134	-0.216	1.00	0.329	-0.332	-0.245
Automaticity	-0.219	-0.011	0.196	-0.134	0.110	0.234	0.443	1.00	0.155	-0.056	0.112	0.247	-0.170	-0.171	-0.103	0.092	0.183	0.158	-0.095
Cybersickness	0.220	0.411	-0.155	0.602**	-0.387	-0.224	0.015	0.042	1.00	0.030	0.296	0.068	-0.006	0.387	0.093	-0.116	0.147	0.381	0.508*
Presence	0.079	-0.261	0.009	-0.129	0.573**	0.632**	-0.021	-0.112	0.014	1.00	0.396	-0.112	-0.045	0.614**	0.223	-0.245	0.120	0.282	0.235
Immersive_propensity	-0.127	0.097	-0.279	0.152	0.254	0.402	0.159	-0.078	0.257	0.501*	1.00	-0.216	-0.479*	0.537*	0.156	-0.041	0.207	0.063	0.266
STAI_State	0.663**	0.223	0.401	0.032	-0.144	0.050	0.430	0.143	-0.059	0.049	-0.177	1.00	0.252	-0.161	0.131	0.226	0.482*	-0.189	-0.236
STAI_Trait	0.628**	0.097	0.478*	0.027	-0.126	-0.061	-0.219	-0.180	-0.020	0.095	-0.381	0.429	1.00	-0.206	-0.097	0.265	0.296	0.173	-0.126
Absorption_trait	-0.070	-0.019	-0.310	0.110	0.094	0.372	-0.027	-0.286	0.392	0.643**	0.579**	-0.029	0.038	1.00	0.065	-0.126	0.142	0.408	0.408
Dissociation_trait	0.119	0.229	-0.086	0.364	0.023	0.083	-0.136	-0.198	0.670**	0.266	0.214	-0.012	-0.075	0.413	1.00	0.221	-0.186	0.125	0.345
Age	0.052	-0.069	-0.049	-0.383	0.129	-0.112	-0.113	0.152	-0.298	-0.153	-0.046	0.153	0.100	-0.081	0.006	1.00	0.241	0.157	-0.329
Sex	0.281	-0.031	0.288	-0.014	-0.028	0.107	0.429	0.128	-0.098	0.165	0.146	0.526*	0.305	0.187	-0.377	0.216	1.00	0.205	-0.184
Prior_VR	0.059	-0.072	-0.232	0.010	0.000	0.076	-0.147	0.096	0.148	0.264	-0.003	-0.186	0.132	0.392	0.019	0.166	0.205	1.00	-0.118
Prior_hypnosis	-0.145	0.291	-0.156	0.474*	-0.163	-0.245	-0.054	-0.144	0.672**	0.162	0.219	-0.199	-0.057	0.415	0.529*	-0.276	-0.184	-0.118	1.00

Table S2. Full correlation matrix of study variables.

The table presents correlations among 19 variables including anxiety, pain, absorption, dissociation, wakefulness/arousal, automaticity, cybersickness, presence, immersive propensity, STAI scores, absorption and dissociation traits, age, sex, prior VR use, and prior hypnosis practice. Pearson correlation coefficients (r) are reported in the lower triangle, and Spearman rank correlation coefficients (ρ) are reported in the upper triangle. Significance levels are indicated as follows: * $p < 0.05$, ** $p < 0.01$, *** $p < 0.001$. The diagonal contains correlations of each variable with itself (1.00).

Variable coding: Sex (Female = 1, Male = 0); Prior VR use (Yes = 1, No = 0); Prior hypnosis practice (Yes = 1, No = 0). Positive coefficients indicate a direct relationship; negative coefficients indicate an inverse relationship. Correlations are exploratory and not corrected for multiple comparisons.

Variable 1	Variable 2	W	p-value	Normality
Anxiety_pre	Anxiety_post	0.574	< .001	No
Anxiety_pre	Pain_pre	0.945	0.304	Yes
Anxiety_pre	Pain_post	0.846	0.005	No
Anxiety_pre	Absorption	0.948	0.339	Yes
Anxiety_pre	Dissociation	0.953	0.408	Yes
Anxiety_pre	Wakefulness/arousal	0.933	0.179	Yes
Anxiety_pre	Automaticity	0.957	0.483	Yes
Anxiety_pre	Perception of time (min.)	0.929	0.150	Yes
Anxiety_pre	Cybersickness	0.796	< .001	No
Anxiety_pre	Presence	0.948	0.343	Yes
Anxiety_pre	Immersive propensity	0.922	0.124	Yes
Anxiety_pre	STAI_State score	0.982	0.959	Yes
Anxiety_pre	STAI_Trait score	0.972	0.820	Yes
Anxiety_pre	Absorption (trait)	0.963	0.639	Yes
Anxiety_pre	Dissociation (trait)	0.845	0.006	No
Anxiety_pre	Age	0.948	0.333	Yes
Anxiety_pre	Sex (F=1, M=0)	0.944	0.289	Yes
Anxiety_pre	Prior_VR use	0.553	< .001	No
Anxiety_pre	Prior_hypnosis practice	0.457	< .001	No
Anxiety_post	Pain_pre	0.590	< .001	No
Anxiety_post	Pain_post	0.619	< .001	No
Anxiety_post	Absorption	0.692	< .001	No
Anxiety_post	Dissociation	0.670	< .001	No
Anxiety_post	Wakefulness/arousal	0.626	< .001	No
Anxiety_post	Automaticity	0.659	< .001	No
Anxiety_post	Perception of time (min.)	0.608	< .001	No
Anxiety_post	Cybersickness	0.599	< .001	No
Anxiety_post	Presence	0.680	< .001	No
Anxiety_post	Immersive propensity	0.691	< .001	No
Anxiety_post	STAI_State score	0.657	< .001	No
Anxiety_post	STAI_Trait score	0.575	< .001	No
Anxiety_post	Absorption (trait)	0.706	< .001	No
Anxiety_post	Dissociation (trait)	0.744	< .001	No
Anxiety_post	Age	0.686	< .001	No
Anxiety_post	Sex (F=1, M=0)	0.696	< .001	No
Anxiety_post	Prior_VR use	0.552	< .001	No
Anxiety_post	Prior_hypnosis practice	0.663	< .001	No
Pain_pre	Pain_post	0.803	< .001	No
Pain_pre	Absorption	0.924	0.119	Yes
Pain_pre	Dissociation	0.762	< .001	No
Pain_pre	Wakefulness/arousal	0.729	< .001	No
Pain_pre	Automaticity	0.904	0.048	Yes
Pain_pre	Perception of time (min.)	0.860	0.008	No
Pain_pre	Cybersickness	0.675	< .001	No
Pain_pre	Presence	0.854	0.006	No
Pain_pre	Immersive propensity	0.925	0.142	Yes
Pain_pre	STAI_State score	0.719	< .001	No
Pain_pre	STAI_Trait score	0.890	0.033	Yes
Pain_pre	Absorption (trait)	0.786	< .001	No
Pain_pre	Dissociation (trait)	0.882	0.023	Yes
Pain_pre	Age	0.868	0.011	No
Pain_pre	Sex (F=1, M=0)	0.748	< .001	No
Pain_pre	Prior_VR use	0.362	< .001	No
Pain_pre	Prior_hypnosis practice	0.421	< .001	No
Pain_post	Absorption	0.920	0.100	Yes
Pain_post	Dissociation	0.807	0.001	No
Pain_post	Wakefulness/arousal	0.780	< .001	No
Pain_post	Automaticity	0.715	< .001	No
Pain_post	Perception of time (min.)	0.890	0.027	Yes
Pain_post	Cybersickness	0.650	< .001	No
Pain_post	Presence	0.866	0.010	No
Pain_post	Immersive propensity	0.786	< .001	No
Pain_post	STAI_State score	0.817	0.002	No
Pain_post	STAI_Trait score	0.736	< .001	No
Pain_post	Absorption (trait)	0.819	0.002	No
Pain_post	Dissociation (trait)	0.789	< .001	No
Pain_post	Age	0.790	< .001	No

Pain_post	Sex (F=1, M=0)	0.779	< .001	No
Pain_post	Prior_VR use	0.473	< .001	No
Pain_post	Prior_hypnosis practice	0.640	< .001	No
Absorption	Dissociation	0.883	0.020	No
Absorption	Wakefulness/arousal	0.940	0.245	Yes
Absorption	Automaticity	0.946	0.308	Yes
Absorption	Perception of time (min.)	0.924	0.121	Yes
Absorption	Cybersickness	0.832	0.003	No
Absorption	Presence	0.943	0.268	Yes
Absorption	Immersive propensity	0.928	0.157	Yes
Absorption	STAI_State score	0.903	0.055	Yes
Absorption	STAI_Trait score	0.921	0.118	Yes
Absorption	Absorption (trait)	0.923	0.129	Yes
Absorption	Dissociation (trait)	0.863	0.011	No
Absorption	Age	0.917	0.087	Yes
Absorption	Sex (F=1, M=0)	0.935	0.192	Yes
Absorption	Prior_VR use	0.515	< .001	No
Absorption	Prior_hypnosis practice	0.895	0.039	Yes
Dissociation	Wakefulness/arousal	0.870	0.012	No
Dissociation	Automaticity	0.929	0.145	Yes
Dissociation	Perception of time (min.)	0.921	0.103	Yes
Dissociation	Cybersickness	0.759	< .001	No
Dissociation	Presence	0.931	0.158	Yes
Dissociation	Immersive propensity	0.901	0.051	Yes
Dissociation	STAI_State score	0.960	0.567	Yes
Dissociation	STAI_Trait score	0.941	0.278	Yes
Dissociation	Absorption (trait)	0.965	0.679	Yes
Dissociation	Dissociation (trait)	0.881	0.022	No
Dissociation	Age	0.974	0.829	Yes
Dissociation	Sex (F=1, M=0)	0.941	0.253	Yes
Dissociation	Prior_VR use	0.511	< .001	No
Dissociation	Prior_hypnosis practice	0.615	< .001	No
Wakefulness/arousal	Automaticity	0.692	< .001	No
Wakefulness/arousal	Perception of time (min.)	0.900	0.041	Yes
Wakefulness/arousal	Cybersickness	0.711	< .001	No
Wakefulness/arousal	Presence	0.894	0.032	Yes
Wakefulness/arousal	Immersive propensity	0.920	0.114	Yes
Wakefulness/arousal	STAI_State score	0.939	0.251	Yes
Wakefulness/arousal	STAI_Trait score	0.968	0.734	Yes
Wakefulness/arousal	Absorption (trait)	0.886	0.028	Yes
Wakefulness/arousal	Dissociation (trait)	0.885	0.026	Yes
Wakefulness/arousal	Age	0.937	0.207	Yes
Wakefulness/arousal	Sex (F=1, M=0)	0.845	0.004	No
Wakefulness/arousal	Prior_VR use	0.488	< .001	No
Wakefulness/arousal	Prior_hypnosis practice	0.490	< .001	No
Automaticity	Perception of time (min.)	0.883	0.020	No
Automaticity	Cybersickness	0.655	< .001	No
Automaticity	Presence	0.949	0.353	Yes
Automaticity	Immersive propensity	0.892	0.034	Yes
Automaticity	STAI_State score	0.913	0.086	Yes
Automaticity	STAI_Trait score	0.971	0.802	Yes
Automaticity	Absorption (trait)	0.955	0.483	Yes
Automaticity	Dissociation (trait)	0.874	0.017	No
Automaticity	Age	0.974	0.829	Yes
Automaticity	Sex (F=1, M=0)	0.903	0.047	Yes
Automaticity	Prior_VR use	0.619	< .001	No
Automaticity	Prior_hypnosis practice	0.490	< .001	No
Perception of time (min.)	Cybersickness	0.752	< .001	No
Perception of time (min.)	Presence	0.921	0.103	Yes
Perception of time (min.)	Immersive propensity	0.918	0.102	Yes
Perception of time (min.)	STAI_State score	0.933	0.197	Yes
Perception of time (min.)	STAI_Trait score	0.945	0.323	Yes
Perception of time (min.)	Absorption (trait)	0.939	0.248	Yes
Perception of time (min.)	Dissociation (trait)	0.884	0.026	Yes
Perception of time (min.)	Age	0.933	0.174	Yes
Perception of time (min.)	Sex (F=1, M=0)	0.904	0.049	Yes
Perception of time (min.)	Prior_VR use	0.426	< .001	No
Perception of time (min.)	Prior_hypnosis practice	0.362	< .001	No

Cybersickness	Presence	0.752	< .001	No
Cybersickness	Immersive propensity	0.628	< .001	No
Cybersickness	STAI_State score	0.650	< .001	No
Cybersickness	STAI_Trait score	0.643	< .001	No
Cybersickness	Absorption (trait)	0.604	< .001	No
Cybersickness	Dissociation (trait)	0.655	< .001	No
Cybersickness	Age	0.705	< .001	No
Cybersickness	Sex (F=1, M=0)	0.713	< .001	No
Cybersickness	Prior_VR use	0.572	< .001	No
Cybersickness	Prior_hypnosis practice	0.631	< .001	No
Presence	Immersive propensity	0.853	0.008	No
Presence	STAI_State score	0.970	0.778	Yes
Presence	STAI_Trait score	0.941	0.274	Yes
Presence	Absorption (trait)	0.934	0.202	Yes
Presence	Dissociation (trait)	0.878	0.020	No
Presence	Age	0.899	0.039	Yes
Presence	Sex (F=1, M=0)	0.923	0.115	Yes
Presence	Prior_VR use	0.547	< .001	No
Presence	Prior_hypnosis practice	0.437	< .001	No
Immersive propensity	STAI_State score	0.935	0.216	Yes
Immersive propensity	STAI_Trait score	0.910	0.074	Yes
Immersive propensity	Absorption (trait)	0.976	0.889	Yes
Immersive propensity	Dissociation (trait)	0.872	0.016	No
Immersive propensity	Age	0.908	0.068	Yes
Immersive propensity	Sex (F=1, M=0)	0.930	0.172	Yes
Immersive propensity	Prior_VR use	0.496	< .001	No
Immersive propensity	Prior_hypnosis practice	0.554	< .001	No
STAI_State score	STAI_Trait score	0.972	0.823	Yes
STAI_State score	Absorption (trait)	0.916	0.095	Yes
STAI_State score	Dissociation (trait)	0.849	0.007	No
STAI_State score	Age	0.969	0.749	Yes
STAI_State score	Sex (F=1, M=0)	0.955	0.476	Yes
STAI_State score	Prior_VR use	0.452	< .001	No
STAI_State score	Prior_hypnosis practice	0.441	< .001	No
STAI_Trait score	Absorption (trait)	0.841	0.005	No
STAI_Trait score	Dissociation (trait)	0.856	0.009	No
STAI_Trait score	Age	0.965	0.683	Yes
STAI_Trait score	Sex (F=1, M=0)	0.960	0.569	Yes
STAI_Trait score	Prior_VR use	0.490	< .001	No
STAI_Trait score	Prior_hypnosis practice	0.463	< .001	No
Absorption (trait)	Dissociation (trait)	0.879	0.021	No
Absorption (trait)	Age	0.977	0.899	Yes
Absorption (trait)	Sex (F=1, M=0)	0.947	0.357	Yes
Absorption (trait)	Prior_VR use	0.529	< .001	No
Absorption (trait)	Prior_hypnosis practice	0.362	< .001	No
Dissociation (trait)	Age	0.819	0.002	No
Dissociation (trait)	Sex (F=1, M=0)	0.819	0.002	No
Dissociation (trait)	Prior_VR use	0.861	0.010	No
Dissociation (trait)	Prior_hypnosis practice	0.772	< .001	No
Age	Sex (F=1, M=0)	0.943	0.278	Yes
Age	Prior_VR use	0.482	< .001	No
Age	Prior_hypnosis practice	0.624	< .001	No
Sex (F=1, M=0)	Prior_VR use	0.362	< .001	No
Sex (F=1, M=0)	Prior_hypnosis practice	0.616	< .001	No
Prior_VR use	Prior_hypnosis practice	0.362	< .001	No

Table S3. Shapiro–Wilk Test for Bivariate Normality of All Study Variables.
Shapiro–Wilk tests were conducted for each pair of variables to assess bivariate normality prior to correlation analyses. For each variable pair, the table reports the Shapiro–Wilk W statistic and its corresponding p-value. A significant p-value (< .05) indicates a deviation from normality. In the final column, normality is coded as “Yes” if the bivariate distribution did not significantly deviate from normality, and “No” if it did. For correlation analyses, Pearson’s r was used for normally distributed variable pairs (Normality = Yes), whereas Spearman’s rho was used when normality was violated (Normality = No).

Variable Abbreviations:

Anxiety_pre = Pre-intervention anxiety score; Anxiety_post = Post-intervention anxiety score; Pain_pre = Pre-intervention pain rating; Pain_post = Post-intervention pain rating; Absorption = State absorption during VRH session; Dissociation = State dissociation during VRH session; Wakefulness/arousal = Level of wakefulness/arousal during VRH session; Automaticity = Automaticity during during; Cybersickness = Total cybersickness score; Presence = Subjective sense of presence during VRH session; Immersive propensity = Immersive tendency (trait); STAI_State score = State anxiety from STAI; STAI_Trait score = Trait anxiety from STAI; Absorption (trait) = Trait absorption (Tellegen); Dissociation (trait) = Trait dissociation (DES-30); Age = Patient age (years); Sex (F=1, M=0) = Patient sex; Prior_VR use (Yes=1, No=0) = Previous VR experience; Prior_hypnosis practice (Yes=1, No=0) = Prior hypnosis practice
